# EHMTI-0312. Long term efficiency and tolerability of greater occipital nerve infiltrations in primary headache disorders

**DOI:** 10.1186/1129-2377-15-S1-G44

**Published:** 2014-09-18

**Authors:** S Lim, T Young

**Affiliations:** 1 The Headache Service, The National Hospital for Neurology & Neurosurgery Queen Square London, London, UK; 2 The Headache Service, The National Hospital for Neurology & Neurosurgery, London, UK

## Introduction

Greater Occipital Nerve (GON) infiltrations with local anaesthetic and steroid have been utilized in primary headache disorders for decades. Despite this we are not aware of significant long-term data of tolerability and effectiveness beyond 26 months.

## Aims and methods

From a 2012 department audit 8 patients under the Headache Service with ongoing GON infiltrations for 3 years or more were identified, with additional data for these patients added up to the present time. Of this cohort, 6 had a diagnosis of chronic migraine (3 with medication-overuse headache, 1 had basilar-type migraine and 1 had additional Familial hemiplegic migraine); 1 had New daily persistent headache (migrainous variant) and 1 had Chronic cluster headache.

## Results

A total of 145 GON infiltrations were delivered to this group over a mean time period of 6 years 2 months (range 4 years to 10 years 11 months).

All patients continued to experience some benefit over this period, of whom 4/8 had responses at the last injection comparable to that after the first injection. Mild adverse reactions included pain, numbness or dizziness following injections. One patient experienced worsening of pre-existing basilar migraine attacks (after their first GON infiltration) and one developed Valsalva headaches within a month of their last GON infiltration.

## Conclusions

Whilst this may be a self-selecting group, we believe this is the longest duration data published on effectiveness and tolerability of GON infiltrations. In some patients at least, retained effectiveness and tolerability of GON infiltration seems possible for more than 6 years.

No conflict of interest.

